# *Helicobacter pylori bab* characterization in clinical isolates from Bhutan, Myanmar, Nepal and Bangladesh

**DOI:** 10.1371/journal.pone.0187225

**Published:** 2017-11-06

**Authors:** Shamshul Ansari, Evariste Tshibangu Kabamba, Pradeep Krishna Shrestha, Hafeza Aftab, Thein Myint, Lotay Tshering, Rabi Prakash Sharma, Nwe Ni, Than Than Aye, Phawinee Subsomwong, Tomohisa Uchida, Thawee Ratanachu-ek, Ratha-korn Vilaichone, Varocha Mahachai, Takashi Matsumoto, Junko Akada, Yoshio Yamaoka

**Affiliations:** 1 Department of Environmental and Preventive Medicine, Oita University Faculty of Medicine, Yufu, Japan; 2 Gastroenterology Department, Maharajgunj Medical Campus, Tribhuvan University Teaching Hospital, Kathmandu, Nepal; 3 Department of Gastroenterology, Dhaka Medical College and Hospital, Dhaka, Bangladesh; 4 Department of Gastroenterology, Yangon General Hospital, Yangon, Myanmar; 5 Department of Surgery, Jigme Dorji Wangchuck National Referral Hospital, Thimphu, Bhutan; 6 Department of Gastroenterology, Mandalay General Hospital and University of Medicine (Mandalay), Mandalay, Myanmar; 7 Department of Gastroenterology, Thingangyun Sanpya General Hospital and University of Medicine (2), Thingangyun, Myanmar; 8 Department of Molecular Pathology, Faculty of Medicine, Oita University, Hasama-machi, Yufu-City, Oita, Japan; 9 Department of Surgery, Rajavithi Hospital, Bangkok, Thailand; 10 Gastroenterology Unit, Department of Medicine, Thammasat University Hospital, Pathum Thani, Thailand; 11 GI and Liver Center, Bangkok Medical Center, Bangkok, Thailand; 12 Department of Medicine-Gastroenterology, Baylor College of Medicine, Houston, TX, United States of America; National Cancer Center, JAPAN

## Abstract

**Background:**

*Helicobacter pylori* BabA is an important outer membrane protein that involves in the attachment to the gastric mucosa and enhances the virulence property of the bacterium. This study was aimed to characterize the *bab* genotypes, to evaluate its association with *cagA*, *vacA* and clinical diseases as well as degree of gastric inflammation.

**Methods:**

*H*. *pylori* isolates from four countries were subjected for the characterization of *bab*. The locus specific forward and *bab* specific reverse primers were used to get the specific products by PCR, which could distinguish the three locus (A, B and C). The histological activities were evaluated according to the Updated Sydney system.

**Result:**

In patients from high risk countries (Bhutan and Myanmar) relatively higher frequencies of strains with *babA*-positivity (91.8% and 90.7%, respectively), *babA* at locus A (98% and 91.2%, respectively) and with single *babA* (96.8% and 91.2%, respectively) were found. Strains with two loci occupied were the most prevalent in Bhutan (84.6%), Myanmar (74.7%), Nepal (58.3%) and Bangladesh (56.9%). The genotype *babA* at locus A*/babB* at locus B*/bab*-negative at locus C (*babA/babB*/-) was the most common genotype isolated from Bhutan (82.7%), Myanmar (58.7%), Nepal (32%) and Bangladesh (31.4%) among all genotypes assessed. This genotype was also associated with the peptic ulcer disease (P = 0.013) when compared to gastritis. *babA*-positive characteristics and the genotype *babA/babB*/- exhibited the enhanced histological activities.

**Conclusions:**

The higher prevalence of virulence associated *babA*-positive characteristics and enhanced histological activities in Bhutan than in Myanmar, Nepal and Bangladesh might partly explain why the peoples in Bhutan are at higher risk for developing severe gastric complications.

## Introduction

The *Helicobacter pylori (H*. *pylori)*, a Gram-negative helical bacterium, is a gastric pathogen that chronically infects at least 50% of the world’s population [1]. The prevalence of the infection ranges from 24.4% in Oceania to 70.1% in Africa [2]. The *H*. *pylori* utilizes various putative virulence factors such as CagA, VacA and outer membrane proteins (OMPs) such as blood group antigen binding adhesin (BabA), sialic acid binding adhesin (SabA) and outer inflammatory protein (OipA) [3–6].

BabA (OMP28 or HopS) is around 75–80 kDa protein with its closely related paralogs; BabB (OMP19 or HopT) and BabC (OMP9 or HopU) [7, 8]. The three *bab* genes, i. e. *babA*, *babB* and *babC* can be found in at least 3 different genomic loci which can be represented by the 3 marker genes. The *bab* gene located downstream of gene *hypD*, *s18* and *hp0318* represents the localization of *bab* gene at locus A, locus B and locus C, respectively in 26695 strain [9]. The recent X-ray structure of BabA revealed three pronged Lewis b (Leb) binding sites, two diversity loops (DL1 and DL2) and one conserved loop (CL2) [10]. The epidemiological studies suggest that the prevalence of *babA*-positive strains tends to differ from different parts of the world [11–14]. In most of the previous studies, the functional status of *babA* was evaluated by using the *babA2* specific primers which can detect the 10-bp deletion in the signal region of *babA* (silent *babA1* and expressed *babA2*) [15]. However, this method is not reliable and is questionable since this frameshift change (*babA1*) was very rare in clinical isolates [16].

The association of BabA-positive status with the increased risk for the development of peptic ulcer diseases (PUDs) has been documented [15, 17]. Although the association between *H*. *pylori* infection and gastric cancer has already been established [18, 19], high infection rate in Asian countries is not always associated with high incidence of gastric cancer [20, 21]. Despite of the high prevalence of *H*. *pylori* infection in some countries show low incidence of gastric cancer while others show high incidence of gastric cancer among Asian countries, the phenomenon has been termed the “Asian enigma” and the age-standardized incidence rate (ASR) of gastric cancer in Asian countries tends to be variable [20, 21]. For example it is low in Bangladesh and Nepal (5.8 and 5.3 cases per 100,000 population per year, respectively), intermediate in Myanmar (11.0 cases per 100,000 population per year) when compared to Bhutan where it is high (17.2 cases per 100,000 population per year) (GLOBOCAN 2012) (http://*globocan*.*iarc*.*fr**)* [22]. Despite of being developing countries with low socio-economic status the incidence of gastric cancer remains variable in these countries [22]. Furthermore, there are none or few published data about the *bab* paralogous genes in relation to their respective genomic locus from the clinical cases of Asian countries [23]. Therefore, this study was aimed to document the prevalence of the *bab* genotypes in clinical strains from high (Bhutan), intermediate (Myanmar) and low risk (Nepal and Bangladesh) populations; to find the association between *bab* genotypes and *cagA*, *vacA* genotypes, to find the association between *bab* genotypes and clinical outcomes, as well as to find the relationship between *bab* genotype and histological activities.

## Methods

### Subjects and biopsy specimens

Dyspeptic patients meeting the inclusion criteria were recruited for the endoscopic survey in Bhutan (Thimphu, Punakha and Wangdu) in 2010, Myanmar (Yangon and Mandalay) in 2011, Nepal (Kathmandu) in 2012 and Bangladesh (Dhaka) in 2014. Patients of more than 16 years old having dyspeptic symptoms were enrolled and those with history of total/partial gastric resection or upper gastrointestinal bleeding or having previous eradication therapy with antibiotics, proton pump inhibitors and bismuth containing compound were excluded from this study. Four gastric biopsies were collected from each patient by experienced endoscopists as described previously [24, 25, 21, 26]. The clinical presentations of gastric ulcer, duodenal ulcer and gastric cancer were identified by endoscopic examination whereas gastritis was identified histologically and gastritis was defined as *H*. *pylori* gastritis in the absence of gastric ulcer, duodenal ulcer or gastric cancer. Gastric cancer was further confirmed by histopathology methods.

### Ethical approval

Written informed consent was obtained from each patient enrolled in this study and the research protocol was approved by Ethics Committee of Jigme Dorji Wangchuk National Referral Hospital in Bhutan, Yangon General Hospital and Mandalay General Hospital in Myanmar, Tribhuvan University Teaching Hospital in Nepal, Bangladesh Medical Research Council in Bangladesh and Oita University Faculty of Medicine in Japan.

### Bacterial strains

The *H*. *pylori* culture and isolation was performed as previously described with minor modification [27]. Briefly, the homogenized biopsy specimens were inoculated onto the commercially available Helicobacter selective agar plate (Nissui Pharmaceutical Co. Ltd. Tokyo, Japan). All the inoculated plates were incubated at 37°C for up to 10 days under microaerophilic condition with 5% CO_2_. Bacterial growth was identified as *H*. *pylori* based on the colony morphology, Gram’s staining and biochemical reactions such as oxidase, catalase and urease [28].

### Polymerase chain reactions and genotyping

**Fig 1:**
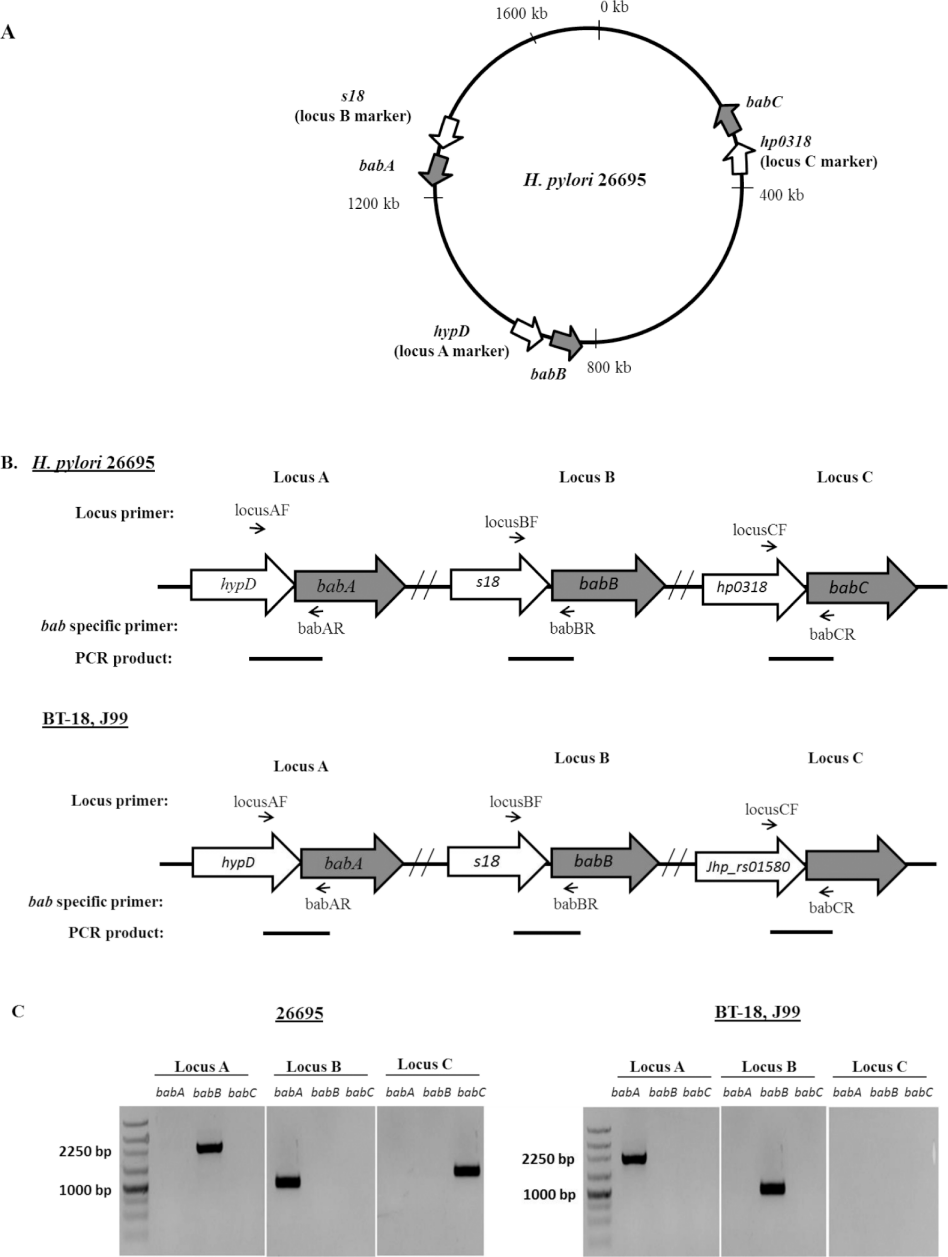
Schematic representation of *bab* genotyping by PCR. **A**- Genomic location of locus marker genes and *bab* genes in 26695. **B**- Locus A, Locus B and Locus C represents the three genomic loci where the *bab* genes are located. The annealing positions of locus specific forward primers are represented as forward arrows. Annealing positions of *bab* specific reverse primers are represented as backward arrows (26695 and Bhutan strain, BT-18 in which *bab*-genotype is similar to J99). **C**- The agarose-gel electrophoresis results showing the positive-bands for *bab* genes (*babA*, *babB* or *babC*) in respective locus in 26695 and BT-18 (similar to J99). The negative result for band in locus C shows the locus is empty (no *bab* gene at locus C in J99 or BT-18).

The *H*. *pylori* isolates grown were subjected for the DNA extraction. Genomic DNA from all clinical isolates and *H*. *pylori* reference strains J99 and 26695 were extracted using commercially available DNA extraction kit (QIAamp DNA Mini Kit; QIAGEN, Valencia, CA, USA) according to the manufacturer’s instruction. The extracted DNA was subjected for the detection of *bab* genes and their respective location by using polymerase chain reactions (PCRs). Locus-specific forward primer (i.e., *hypD* primer for locus A, *s18* primer for locus B or *Hp1-AS* primer for locus C) and *bab*-specific reverse primers were used for PCRs to investigate the presence or absence of *babA*, *babB* or *babC*, respectively (**Fig 1B**) [29, 30, 23]. All PCR primers used for genotyping were based on the DNA sequences of *H*. *pylori* strains J99 and 26695 (**Table A in S1 File**). All PCR reactions were performed using standard concentrations of reagents and under following conditions: 94°C for 5 min; 40 cycles of 94°C for 30 s, 56.5°C for 30 s and 72°C for 2 min, followed by 72°C for 5 min [29]. After PCR was completed, the products were carried out for electrophoresis using 1% agarose gel stained with ethidium bromide (Gibco, BRL, San Francisco, CA, USA). The expected PCR products were 2.1–2.6 kb if the *babA* or *babB* or *babC* gene was located at locus A, 1.0–1.5 kb if *babA* or *babB* or *babC* gene was located at locus B or around 1.5 kb if *babA* or *babB* or *babC* gene was located at locus C. Previously published data regarding *cagA* and *vacA* status/genotypes were used for comparison with *bab* genotypes [24, 25, 21, 26].

### Genotype designation

The *bab* gene (*babA*, *babB* or *babC*) positive status in respective locus (A, B or C) was designated as *babA* or *babB* or *babC* in respective locus separated by slash (/). For example, the strains showing *babA*-positive status with locus A specific primer, *babB*-positive status with locus B specific primer and *babC*-positive status with locus C specific primer were designated as *babA/babB/babC* genotype. Similarly, any *bab* gene-negative status with any locus specific primer was designated by en dash (-). For example; *babB*-positive status with locus A specific primer, *babA*-negative status with locus B specific primer and *babC-*positive status with locus C specific primer was designated as *babB/-/babC*.

### Histological examinations

Biopsy specimens for histological examination were fixed in 10% formalin and embedded in paraffin. Serial tissue sections were cut, spread on glass slides and stained with hematoxylin and eosin and May-Grunwald Giemsa stains. The tissue sections were also subjected for the immunohistochemistry (IHC) examination as described previously [31]. Briefly, the tissue sections were retrieved for antigen and inactivated for endogenous peroxidase activity. The tissue sections were incubated with *H*. *pylori* antibody (DAKO, Glostrup, Denmark) at 4°C for overnight. The sections were washed and incubated with biotinylated goat anti-rabbit IgG (Nichirei Co., Tokyo, Japan) followed by incubation with a solution of avidin-conjugated horseradish peroxidase (Vectastain Elite ABC kit, Vector Laboratories Inc, Burlingame, CA, USA). Then H_2_O_2_/diaminobenzidine substrate solution was used to detect the peroxidase activity. All sections were subjected for Giemsa staining. After staining all slides were observed by same pathologist (TU) without prior knowledge of patients’ information to exclude the possible bias.

### Determination of gastritis stage

Stained tissue sections were evaluated for degree of inflammation, neutrophil infiltration, monocyte infiltration, atrophy, intestinal metaplasia and bacterial density according to the Updated Sydney System [32]. The tissue was classified into four grades: 0, normal; 1, mild; 2, moderate; and 3, marked [32]. The gastritis stage was also assessed based on the topographic locations in the antrum and corpus, according to the Operative Link on Gastritis Assessment (OLGA) system [33].

### Statistical analysis

The association between discrete variables such as *bab* genotypes, *cagA*, *vacA* genotypes, and clinical outcomes were analyzed by using Pearson’s Chi-square test. In case the expected frequencies in contingency tables were below 5, Fisher’s exact test was used. Odd ratio (OR) and 95% confidence interval (CI) were determined using Epi-info 3.5.4 software. The association between the *babA* characteristics and histological grades for neutrophil infiltration, monocyte infiltration, intestinal metaplasia, atrophy, and *H*. *pylori* density were compared using Pearson’s Chi-square or Fisher’s exact test and Mann Whitney test using R statistics software version 1.35. A P value less than 0.05 was considered statistically significant.

## Results

A total of 903 patients who received endoscopy examination in four countries were enrolled: Bhutan (n = 372), Myanmar (n = 252), Nepal (n = 146) and Bangladesh (n = 133) as described in our previous publications [24, 25, 21, 26]. In this study we included the cases with positive bacterial growth from these countries; 208 from Bhutan, 75 from Myanmar, 40 from Nepal and 51 from Bangladesh. Since the cases enrolled were collected during 2–5 days survey, we could collect only few cases of PUD and gastric cancer. Detailed information has been presented in **Table 1**. We also included 32 Nepal strains from our laboratory stocks for the *bab* genotypes evaluation; however their detailed background information were not confirmed. Therefore, a total of 374 strains were evaluated for *cagA*, *vacA*, clinical outcomes and histological activities and 406 strains including additional 32 Nepal strains were subjected for *bab* genotypes characterization.

**Table 1:** The characteristics of *H*. *pylori* positive cases.

	Total (374)	Bhutan (208)	Myanmar (75)	Nepal (40)	Bangladesh (51)
**Mean age**	37.9 ± 13.6	36.4 ± 13.8	40.7 ± 11.5	44.6 ± 15.8	35.0 ± 11.7
**Male**^**†**^	167 (44.6)	97 (46.6)	30 (40.0)	15 (37.5)	25 (49.0)
**Female**^**‡**^	207 (55.4)	111 (53.4)	45 (60.0)	25 (62.5)	26 (51.0)
**Gastritis**	312 (83.4)	164 (78.8)	67 (89.3)	33 (82.5)	48 (94.1)
**Peptic ulcer**	51 (13.6)	40 (19.2)	4 (5.4)	4 (10.0)	3 (5.9)
**Gastric cancer**	5 (1.4)	1 (0.5)	1 (1.3)	3 (7.5)	0
**Unclear diagnosis**	6 (1.6)	3 (1.5)	3 (4.0)	0	0

Note: the figures indicate the number (percentage)

†- indicates no significant differences of male between countries and

‡- indicates no significant differences of female between countries.

### Status of *babA*-positivity

As *babA*-positive status has been linked with the risk for the development of severe gastro-intestinal diseases [34], we first analyzed the *babA*-positive status in four countries. The prevalence of strains with *babA*-positive status was significantly higher in high to moderate risk populations for gastric cancer (Bhutan and Myanmar) than the low risk populations (Nepal and Bangladesh) (**Table 2**). The prevalence of strains with *babA* at locus A and strains with single *babA* also showed the similar patterns (**Table 2**).

**Table 2:** *babA* characteristics in different countries.

	Total (406)	Bhutan (208)	Myanmar (75)	Nepal (72)^a^	Bangladesh (51)
***babA*-positive**	352 (86.7)	191 (91.8)†•	68 (90.7)§	57 (79.2)	36 (70.6)
***babA*-negative**	54 (13.3)	17 (8.2)	7 (9.3)	15 (20.8)	15 (29.4)
	**Combined (352)**^**b**^	**Bhutan (191)**^**b**^	**Myanmar (68)**^**b**^	**Nepal (57)**^**b**^	**Bangladesh (36)**^**b**^
***babA* at locus A**	320 (91.0)	189 (99.0)#†•	62 (91.2)‡§	42 (73.7)	27 (75.0)
***babA* at locus B**	38 (10.8)	2 (1.0)	11 (16.2)#	16 (28.1)†	9 (25.0)•
***babA* at locus C**	25 (7.1)	6 (3.1)	1 (1.5)‡	11 (19.3)†	7 (19.4)
	**Combined (352)**	**Bhutan (191)**	**Myanmar (68)**	**Nepal (57)**	**Bangladesh (36)**
**Single *babA-*positive**	323 (91.8)	185 (96.8)†•	62 (91.2)‡§	45 (79.0)	31 (86.0)
**Multiple *babA-*positive**	29 (8.2)	6 (3.2)	6 (8.8)	12 (21.0)†	5 (14.0)

Note: the figure indicates number (percentage)

# indicates the significant level between Bhutan and Myanmar

† indicates significant level between Bhutan and Nepal

• indicates the significant level between Bhutan and Bangladesh

‡ indicates the significant level between Myanmar and Nepal

§ indicates significant level between Myanmar and Bangladesh

a- we also included 32 strains from Nepal from our laboratory stocks for the *bab* genotypes evaluation; however their detailed background information were not confirmed.

b- the cases with *babA*-negative were excluded and multiple *babA*-positive were considered positive for two or three loci; example e.g., single strain with 2 *babA*-positive for locus A and locus B was considered positive for both *babA* at locus A and *babA* at locus B

### Prevalence of *babA* with *cagA*, *vacA* s and *vacA* m genotypes

In many previous studies, there were reports that *babA*-positive status was closely related to *cagA*-positive/*vacA* s1 status [23, 35]. However, there are no reports about the relation in *H*. *pylori* from four countries investigated in this study. As for the data for *cagA* and *vacA* genotypes, we used our previous data [24, 25, 21, 26]. In agreement with previous studies, the *babA*-positive status was overall significantly prevalent in strains with *cagA*-positive (P <0.000), *vacA* s1 (P <0.000) and *vacA* m1 (P = 0.014) type status; however individually the relation was found only in Bangladesh strains **(Table 3)**, probably due to small number of *cagA*-negative and/or *vacA* s2 status in other countries.

**Table 3:** *babA* positive status in relation to other virulence factors.

	Total			Bhutan			Myanmar			Nepal			Bangladesh		
	N	*babA* (+)	P	N	*babA* (+)	P	N	*babA* (+)	P	N	*babA* (+)	P	N	*babA* (+)	P
***cagA* (+)**	345	310 (89.8)	**<0.000**	204	188 (92.2)	NS	64	59 (92.2)	NS	39	30 (77.0)	NS	38	33 (86.8)	**<0.000**
***cagA* (-)**	27	15 (55.6)		4	4 (100)		9	7 (77.8)		1	1 (100)		13	3 (23.0)	
***vacA* s1**	358	323 (90.2)	**<0.000**	208	192 (92.3)	NA	71	65 (91.5)	NS	38	31 (81.6)	**0.046**	41	35 (85.4)	**<0.000**
***vacA* s2**	14	2 (14.3)		0	0		2	1 (50.0)		2	0 (0.0)		10	1 (10.0)	
***vacA* m1**	274	245 (89.4)	**0.014**	159	142 (89.3)	NS	62	56 (90.3)	NS	25	21 (84.0)	NS	28	26 (92.8)	**<0.000**
***vacA* m2**	98	78 (79.6)		49	48 (98.0)		11	10 (91.0)		15	10 (66.7)		23	10 (43.5)	
***vacA* s1m1**	275	248 (90.2)	NS	159	142 (89.3)	NS	62	56 (90.3)	NS	24	21 (87.5)	NS	29	27 (93.1)	**0.05**
***vacA* s1m2**	83	75 (90.4)		49	48 (98.0)		9	9 (100)		14	10 (71.4)		12	8 (66.7)	

Note: the figure indicates the number (percentage), **NS**- not significant, **NA**- not applicable

### *bab* genotype distribution

Among the total strains analyzed for *bab* genotyping, 98.5% (400/406) of isolates contained at least one *bab* gene (*babA* and/or *babB* and/or *babC*). The strains were divided into 4 groups based on the *bab* genes found on 3 different loci; one locus occupied, two loci occupied, three loci occupied and all three loci empty (-/-/-). Among one locus occupied group, the genotypes -/-/*babA* and -/*babA*/- were unique genotypes found only among Nepal strains whereas the genotype *babC*/-/- was unique only among Bangladesh strains (**Table 4**). Two loci were occupied by *bab* genes in majority of strains; 84.6% (176/208), 74.7% (56/75) 58.3% (42/72) and 56.9% (29/51) among Bhutan, Myanmar, Nepal and Bangladesh, respectively. The prevalence of *babA/babB*/- genotype was significantly higher in Bhutan than others (P <0.000 for each) and in Myanmar than Nepal (P = 0.001) and Bangladesh (P = 0.003) strains. Occupation of all three loci by *bab* genes was most prevalent in Nepal strains (21/72; 29.2%) and least prevalent in Bhutan strains (6/208; 2.8%). All three loci occupied with *babA* gene (*babA/babA/babA)* was uniquely found only from Bangladesh strains (2/51; 4.0%) (**Table 4**).

**Table 4:** Distribution of *bab* genotypes in Bhutan, Myanmar, Nepal and Bangladesh.

Locus A	Locus B	Locus C	Total (406)	Bhutan (208)	Myanmar (75)	Nepal (72)	Bangladesh (51)
**One locus occupied**							
*babA*	*-*	*-*	27 (6.6)	11 (5.3)	11 (14.7)#	3 (4.1)	2 (4.0)
*-*	*babB*	*-*	24 (5.9)	14 (6.7)	3 (4.0)	3 (4.1)	4 (7.8)
*-*	*-*	*babA*	1 (0.25)	0	0	1 (1.4)	0
*-*	*babA*	*-*	1 (0.25)	0	0	1 (1.4)	0
*babC*	*-*	*-*	1 (0.25)	0	0	0	1 (2.0)
**One locus occupied, total**			**54 (13.3)**	**25 (12.0)**	**14 (18.7)**	**8 (11.1%)**	**7 (13.7)**
**Two loci occupied**							
*babA*	*babB*	*-*	255 (62.8)	172 (82.7)†#•	44 (58.7)‡§	23 (32.0)	16 (31.4)
*babB*	*babB*	*-*	12 (2.9)	1 (0.5)	2 (2.7)	6 (8.3)†	3 (5.9)•
*babA*	*babA*	*-*	12 (2.9)	1 (0.5)	6 (8.0)#	4 (5.6)†	1 (2.0)
*babB*	*babA*	*-*	11 (2.7)	1 (0.5)	3 (4.0)	3 (4.1)	4 (7.8)•
*babB*	*-*	*babA*	1 (0.25)	0	0	1 (1.4)	0
*babA*	*-*	*babA*	1 (0.25)	0	0	1 (1.4)	0
*-*	*babA*	*babB*	1 (0.25)	0	0	1 (1.4)	0
*-*	*babB*	*babB*	1 (0.25)	0	0	1 (1.4)	0
*babB*	*-*	*babB*	1 (0.25)	0	0	1 (1.4)	0
*babC*	*babB*	*-*	7 (1.7)	1 (0.5)	0	1 (1.4)	5 (10.0)•
*babC*	*babA*	*-*	1 (0.25)	0	1 (1.3)	0	0
**Two loci occupied, total**			**303 (74.6)**	**176 (84.6)**	**56 (74.7)**	**42 (58.3)**	**29 (56.9)**
**All 3 loci occupied**							
*babA*	*babB*	*babB*	10 (2.5)	0	1 (1.3)	5 (7)	4 (7.8)
*babA*	*babB*	*babA*	10 (2.5)	5 (2.4)	0	4 (5.6)	1 (2.0)
*babB*	*babA*	*babB*	5 (1.2)	0	0	4 (5.6)	1 (2.0)
*babB*	*babB*	*babA*	8 (2.0)	1 (0.5)	1 (1.3)	2 (4.1)	4 (7.8)•
*babA*	*babA*	*babA*	2 (0.5)	0	0	0	2 (4.0)
*babC*	*babB*	*babC*	1 (0.25)	0	0	1 (1.4)	0
*babA*	*babA*	*babB*	2 (0.5)	0	0	1 (1.4)	1 (2.0)
*babB*	*babB*	*babB*	1 (0.25)	0	0	1 (1.4)	0
*babC*	*babB*	*babA*	1 (0.25)	0	0	1 (1.4)	0
*babA*	*babA*	*babC*	1 (0.25)	0	0	1 (1.4)	0
*babB*	*babA*	*babA*	1 (0.25)	0	0	1 (1.4)	0
*babB*	*babA*	*babC*	1 (0.25)	0	1 (1.3)	0	0
**All 3 loci occupied, total**			**43 (10.6)**	**6 (2.8)**	**3 (4.0)**	**21 (29.2)**	**13 (25.5)**
**All 3 loci empty**							
-	-	-	6 (1.5)	1 (0.5)	2 (2.6)	1 (1.4)	2 (4.0)
**All 3 loci empty, total**			**6 (1.5)**	**1 (0.5)**	**2 (2.6)**	**1 (1.4)**	**2 (4.0)**

Note: the figure indicates number and (percentage)

# indicates the significant level between Bhutan and Myanmar

† indicates significant level between Bhutan and Nepal

• indicates the significant level between Bhutan and Bangladesh

‡ indicates the significant level between Myanmar and Nepal

§ indicates significant level between Myanmar and Bangladesh.

### *babA* characteristics and clinical diseases

The association of *babA*-positive status with the development of PUD and gastric cancer has been reported previously [15]. As expected, the overall *babA*-positive status was associated with the development of PUD (P = 0.034) when compared with gastritis (**Table B in S1 File**). When analyzed individually, this difference was only observed in Bhutan, but without statistically significant level (P = 0.115 and OR = 4.22). Further analysis showed that there were no associations between the clinical outcomes and *babA* locus (**Table C in S1 File**) or the number of *babA* (**Table D in S1 File**). Due to the low number of gastric cancer cases (n = 5) we could not perform the analyses; however all gastric cancer cases were infected with *babA*-positive strains and harboring single *babA* (**Tables B and D in S1 File**).

### *babA*-positive status and histological activities

For histological analysis we excluded PUD and gastric cancer cases because the gastric mucosa of PUD patient is typically associated with enhanced antral inflammation with corpus sparing, whereas that of gastric cancer patient is generally atrophic which can potentially bias the histological analyses. Therefore, we performed the histological analysis on gastritis cases only.

**Fig 2:**
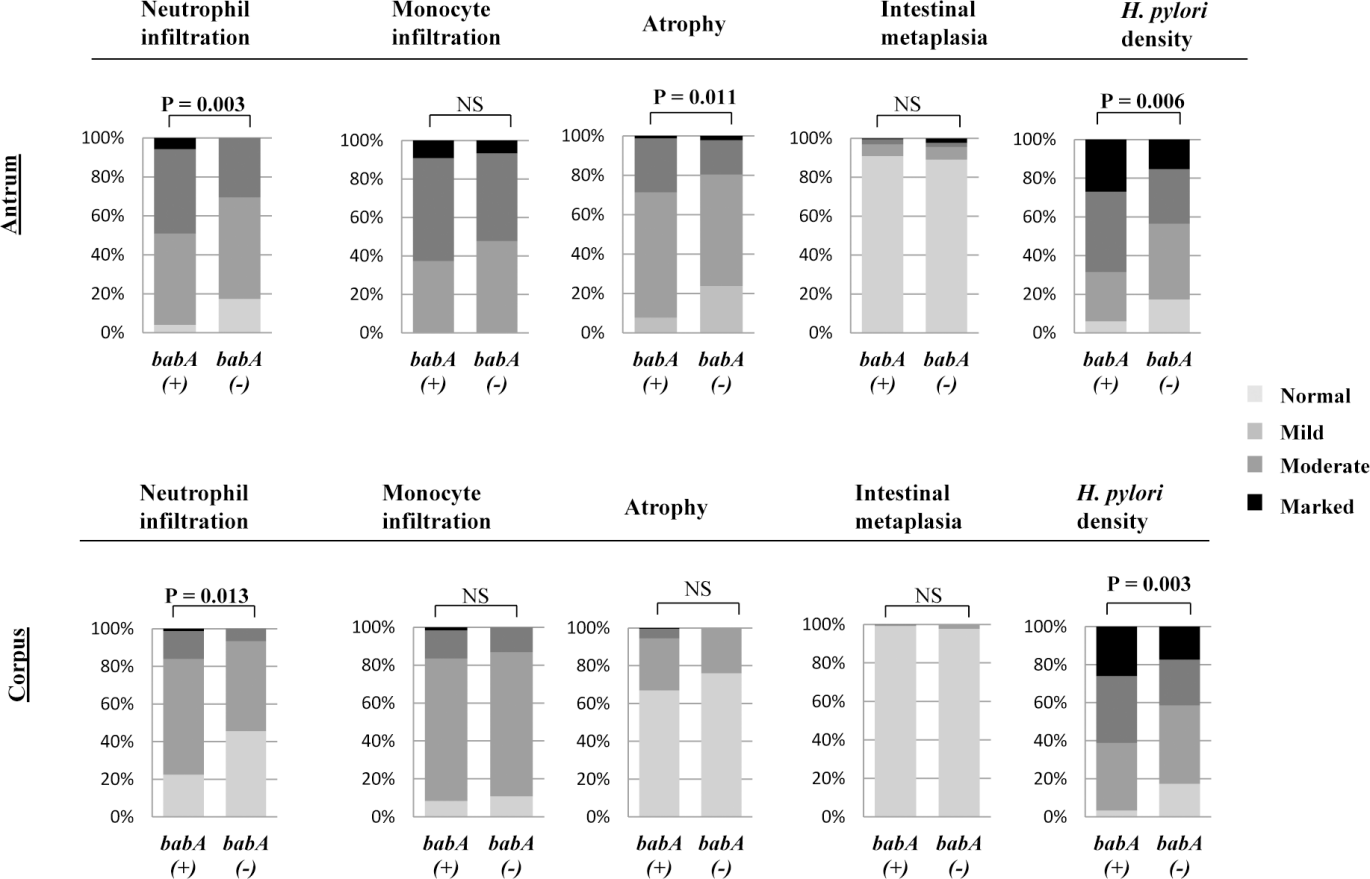
Histological activities and the *H*. *pylori babA*-positive and *babA*-negative status in overall strains from four countries.

**Fig 2** depicts the histological activities according to the *H*. *pylori babA*-positive and negative status in overall strains from four countries. Overall patients infected with *babA-*positive strains showed significantly higher neutrophil infiltration activity in the antrum as well as in the corpus (P = 0.003 and P = 0.013 respectively), atrophy activity in the antrum (P = 0.011) and *H*. *pylori* density in the antrum as well as in the corpus (P = 0.006 and P = 0.003, respectively) than those with *babA*-negative strains (**Fig 2).** The detailed data analyzed individually are shown in **S1 Fig**.

### Genomic location of *babA* and histological activities

As we found that the strains with *babA* at locus A were more prevalent in Bhutan than other countries, we next sought to determine if the *babA* located at locus A could enhance the histological activity higher than strains with *babA* at other locus. As shown in **S2 Fig** which elaborates the histological activities according to the genomic location of *babA*, overall as well as in each country individually we did not get significant association. However, the distribution showed the non-significant greater activities when *babA* was found at locus A. In Bhutan all strains but one contained *babA* at locus A, thus we could not evaluate.

### Number of *babA* and histological activities

**Fig 3:**
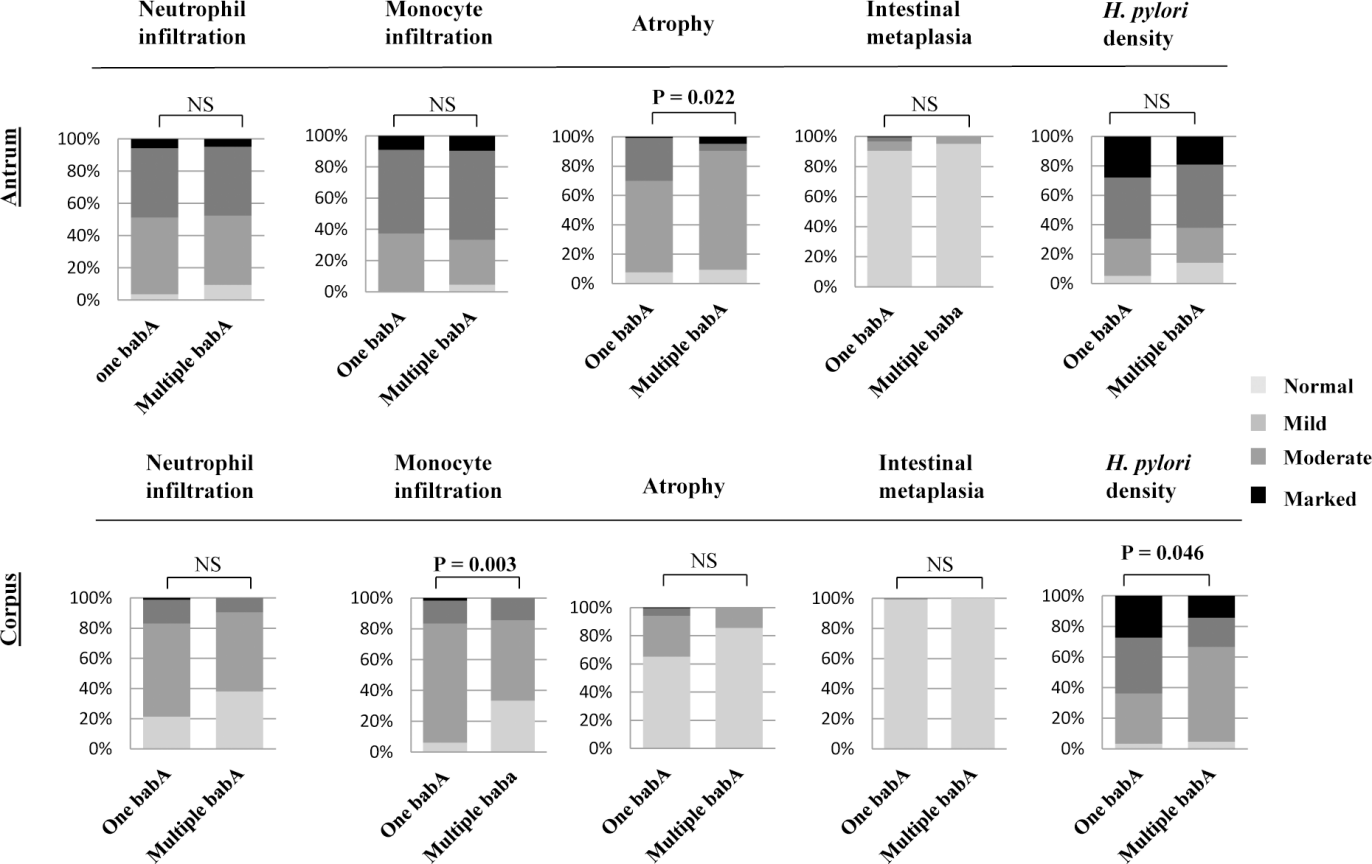
Number of *babA* and histological activities. One *babA* versus multiple *babA* and histological activities in overall strains from four countries.

As elaborated in **Fig 3,** our analysis of the histological activities according to the number of *babA* revealed that overall, the patients infected with strains with single *babA*-positive status exhibited enhanced histological activities in the antrum for atrophy (P = 0.022) and in the corpus for monocyte infiltration (P = 0.003), and *H*. *pylori* density (P = 0.046) than those with multiple *babA*. Individual analysis showed significant differences in the corpus by Bhutanese strains (monocyte infiltration; P = 0.010 and *H*. *pylori* density; P = 0.046) and Nepalese strains (neutrophil infiltration; P = 0.043 and monocyte infiltration; P = 0.002). In contrast, in Bangladesh the patients infected with strains with multiple *babA* exhibited greater histological activity in the antrum than one *babA* strains (monocyte infiltration; P = 0.025). In Myanmar no significant differences in histological activities were observed (**S3 Fig)**.

### Number of locus occupied and histological activities

**Fig 4:**
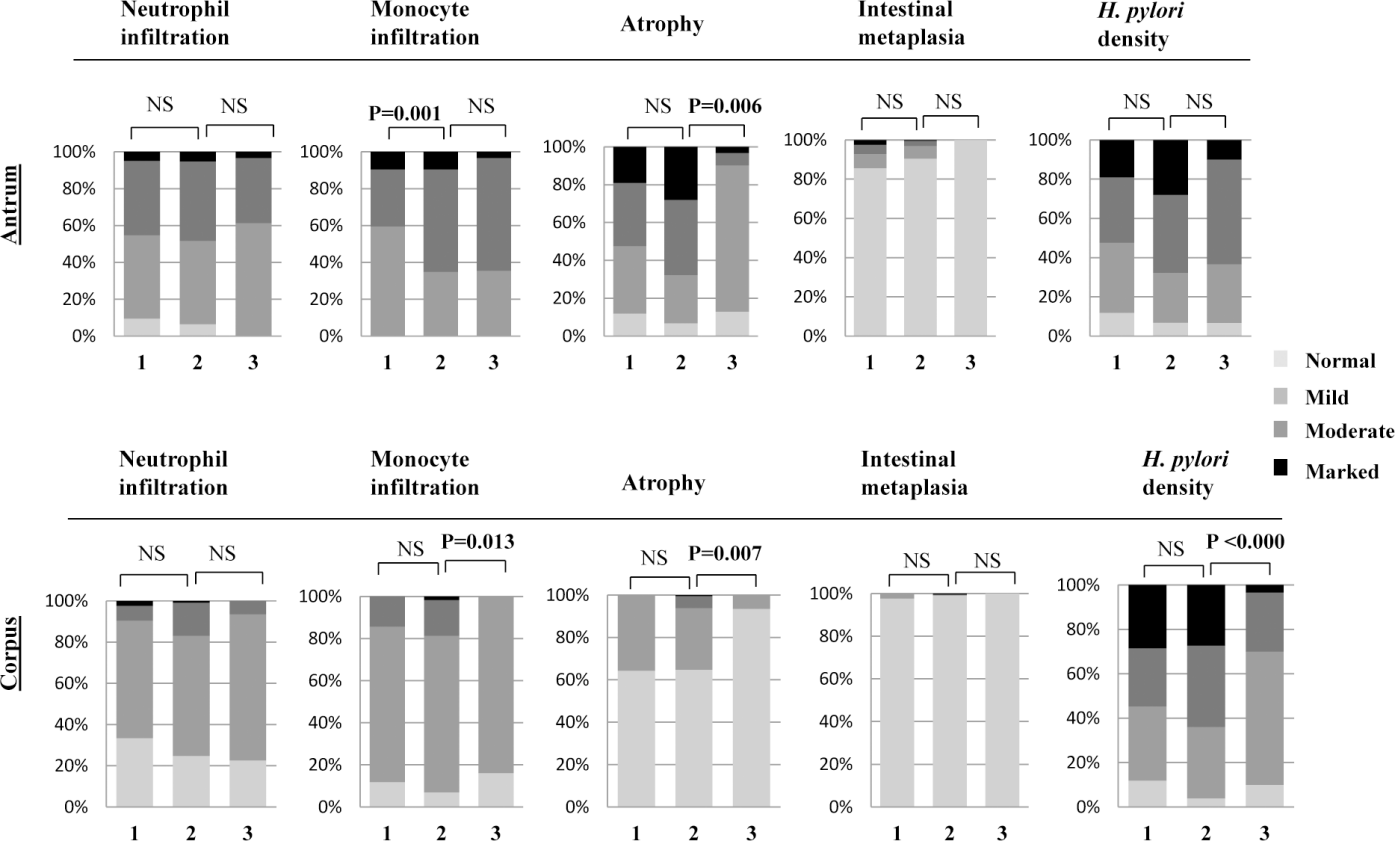
Number of locus occupied and histological activities in overall strains from four countries. 1-one locus occupied, 2-two loci occupied, 3- three loci occupied.

In the present study we found that the strains with two loci occupied were the most prevalent in all the countries and its prevalence was significantly higher in Bhutan (than others) and in Myanmar (than Nepal and Bangladesh). We analyzed whether the patients infected with strains with two loci occupied could execute higher histological activities than those with one locus occupied and three loci occupied. **Fig 4** shows the histological activities according to the number of locus occupied in overall strains analyzed. In our study, the overall patients infected with strains with two loci occupied exhibited greater histological activities than those with one locus occupied (monocyte infiltration in the antrum; P = 0.001) and three loci occupied (atrophy in the antrum and corpus; P = 0.006 and P = 0.007 respectively, monocyte infiltration in the corpus, P = 0.013 and *H*. *pylori* density in the corpus; P <0.000) (**Fig 4**). Individual analysis showed greater histological activities in the antrum with two loci occupied (monocyte infiltration; P = 0.024 and atrophy; P <0.000) than with one locus occupied in Bhutanese strains (**S4 Fig**).

### Clinical diseases and histological activities according to *H*. *pylori bab* genotypes

**Fig 5:**
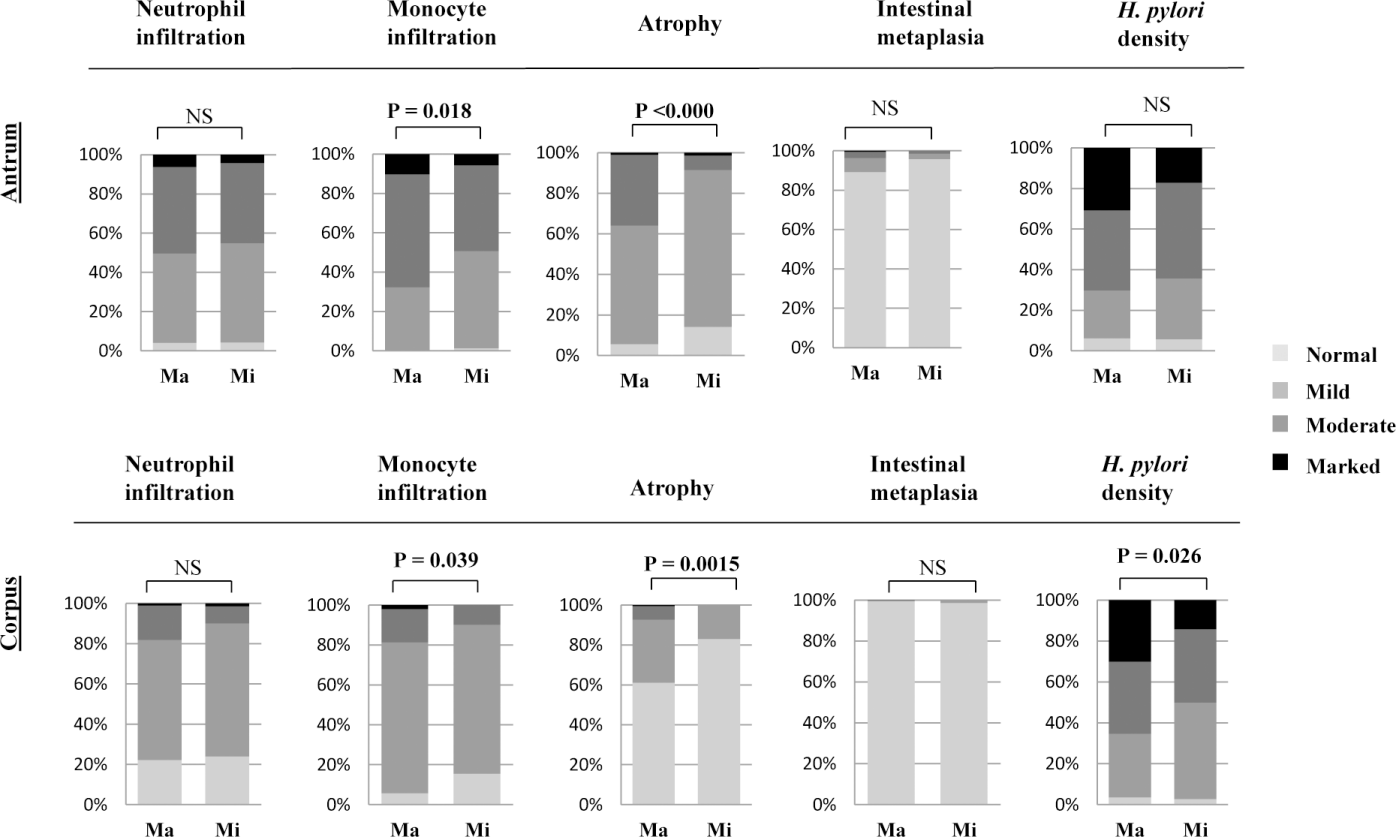
Comparison of histological activities according to the major genotype (*babA/babB/-)* and other genotype with *babA*-positive status (minor genotypes) in overall strains from four countries. Ma-major genotype and Mi-minor genotypes.

Among all genotypes assessed, the genotype *babA/babB/-* was significantly associated (P = 0.013) with the PUD progression when compared with gastritis. The genotypes *babC/babB/babA* and *-/babA/-* were uniquely isolated from gastric cancer cases only (**Table E in S1 File**). The prevalence of the genotype *babA/babB/-* was significantly greater in Bhutan (than other countries) and in Myanmar (than Nepal and Bangladesh) (**Table 4**), suggesting that the strains with this genotype are more virulent than others. We therefore analyzed the histological activities exhibited by strains with *babA/babB/-* genotype (major genotype) over other genotypes with *babA* (minor genotypes). As shown in **Fig 5** in the overall patients infected, the strains with major genotype (*babA/babB/-*) exhibited enhanced histological activities for monocyte infiltration (P = 0.018 in the antrum and P = 0.039 in the corpus), atrophy (P <0.000 in the antrum and P = 0.0015 in the corpus) and *H*. *pylori* density (P = 0.026 in the corpus) than other genotypes with *babA*. Individual analysis showed significant difference, in the antrum (monocyte infiltration; P = 0.002 and atrophy; P = 0.001) and in the corpus (monocyte infiltration; P = 0.003) by Bhutanese strains and in the antrum (monocyte infiltration; P = 0.040) by Myanmar strains (**S5 Fig**).

The genotype *babA/babB/-* also exhibited elevated histological scores when compared with genotypes replacing genes at locus A and locus B such as *babA/-/-*, *-/babB/-*, *babA/babA/-*, *babB/babA/-* and *babB/babB/-* **(Table F in S1 File**).

### Other virulence factors in major (*babA/babB/-)* and minor genotypes (other genotypes with *babA*-positive status)

As the major genotype (*babA/babB/-)* exhibited the enhanced histological activities over minor genotypes. Next we sought to evaluate the prevalence of other virulence factors such as *cagA*, *vacA* s and m genotypes in major and minor genotypes. In overall as well as in individual countries, the major and minor genotypes did not significantly harbor the virulence factors such as *cagA*, *vacA* s and m genotypes **(Table G in S1 File).**

## Discussions

In Asian countries, the epidemiological data suggest a high prevalence of *H*. *pylori* infection with correspondingly variable incidence of gastric cancer. A sero-epidemiological study conducted in Bangladesh reported the detection of anti-VacA antibody in 85% of children suggesting the high prevalence of VacA producing *H*. *pylori*; though there is low incidence of gastric cancer [20]. The strains expressing BabA are classified into “specialists” or “generalists”, depending on their ability to bind with the glycans in blood group O only or with blood group A, B, and O antigens [36]. Therefore, many studies suggest the association of specific bacterial genotype with gastric cancer development despite of the high *H*. *pylori* infection [20]. Most of the previous studies focusing on *babA2* detection do not reliably reflect the BabA functional status [37], furthermore there are only few studies focusing on the different *bab* genes in respect to their genomic location [9, 23]. The present study evaluated the *bab* genes at three different genomic loci to characterize the *bab* genotypes in strains from diverse risk countries.

The genetic variability in outer membrane proteins is one of the vital means to aid in the survival benefits [38]. *H*. *pylori babA*-positive status has been linked with the development of severe complications and more efficiently when it is found at locus A [36]. In this study, the strains with *babA*-positive status and at locus A were more prevalent in Bhutan and moderately prevalent in Myanmar where gastric cancer is high and moderate respectively reflects the importance of characteristics of *babA* in the development of severe diseases. The possible explanation could be the differences in the gastric ecological niches in different countries. Our finding of histological activities was in accordance with the explanation that *H*. *pylori babA* located at locus A was expressed more efficiently and binds with Lewis blood group (Leb) antigen on gastric epithelium leading to the inflammation [36]. Chronic inflammation, atrophy and intestinal metaplasia have been suggested as the precancerous lesions according to the Correa model [39]. In the present study, we reported that the strains with single *babA*-positive status were more prevalent in high risk countries. These strains also exhibited higher histological activities for atrophy in the antrum and chronic inflammation, and *H*. *pylori* density in the corpus. The possible link between the presence of single *babA* and higher histological activities remains to be determined. However, one possible explanation could be that the ancestral strains containing single *babA* at locus A is adapted via gene conversion in adverse environmental niche may contain multiple *babA*, nonetheless; these strains in adverse condition may not efficiently express the BabA. In Bangladesh there was higher activity for chronic inflammation in the antrum by strains with multiple *babA*-positive status. The host genetic difference or environmental factors seem to play the possible role for higher histological activity with multiple *babA* in Bangladesh. It has been reported that complement receptor-3 (CR-3) which is the potent activator of neutrophils and monocytes are involved in the enhanced histological activities [40]. In a study, the *babA*-positivity was found to up-regulate the pathway which is involved in CR-induced neutrophil activation in antrum [41]. These data together suggests the genetic differences in the CR gene may partly explain the higher histological activities by multiple *babA*-positive strains in antral region in Bangladesh.

Despite of the fact that there are several *bab* genotypes reported [9], we found that the strains with two loci occupied; *babA* at locus A and *babB* at locus B with empty locus C (genotype *babA/babB/-)*, are the most common in each countries which is in consistent with the reports of other authors [23, 9]. Interestingly this data suggests that in ancestral strains *babA* was located at locus A and *babB* was located at locus B and gene conversion has resulted in different minor genotypes as the bacteria has to adapt for survival in diverse environmental niche. We found the higher prevalence of the genotype *babA/babB/-* in Bhutan and in Myanmar than in Nepal and Bangladesh when compared with cross-country connecting its possible role in the development of chronic diseases. The enhanced histological activities such as chronic inflammation and atrophy exhibited by this genotype also suggests that in addition to the occupation of locus A with *babA* and locus B with *babB* together with empty locus C aids in its strength to involve more efficiently for the gastric inflammation. The presence of *babA* or *babB* gene at locus C which probably increases the possibility of gene recombination with *bab* genes at locus A and locus B rendering non-functional synthesis of protein may partly explain why the strains with empty locus C seem more virulent.

It is well known that the bacterial, host and environmental factors affect the clinical outcomes [42] and in a given populous region the presence of virulence genes and its polymorphisms play the key role in the pathogenicity [3]. Thus, in our study, we reported the higher prevalence of strains with *babA* characteristics and concurrently enhanced histological activities in high risk populations. The lower prevalence of gastric cancer in Nepal and Bangladesh despite of the higher prevalence of *H*. *pylori* infection; however, cannot fully depend on the genetic diversity of bacterial strain. In our previous study, we evaluated the host RAD51 G135C polymorphism as the important predictor for the gastric cancer in *H*. *pylori* infected patients in Bhutan [43]. Thus, specific host gene polymorphism and environmental factors also account, in part, for gastric cancer risk in Bhutan and perhaps in Myanmar also.

In conclusion, the results of our study of *babA* characteristics indicate that the genotypes with specific *babA* virulence determinants being more prevalent in a high risk population for gastric cancer compared to low risk population could be at least a partial strategy explaining for the ‘Asian enigma’ why in some countries there is low incidence of gastric cancer despite of the high prevalence of *H*. *pylori* infection.

## Supporting information

S1 Fig *H*. *pylori babA*-positive status and histological activities. (DOCX)

S2 Fig Genomic location of *babA* and histological activities. A- *babA* at locus A and B- *babA* at other locus than A. In Bhutan all strains but one contained *babA* at locus A, thus we could not evaluate.
(DOCX)

S3 Fig Number of *babA* and histological activities. (DOCX)

S4 Fig Number of locus occupied and histological activities. 1-one locus occupied, 2- two loci occupied and 3- three loci occupied. In Bhutan only 5 strains were with three loci occupied, in Myanmar only 3 strains were with three loci occupied, in Nepal only 2 strains were with one locus occupied and in Bangladesh there were only 6 strains with one locus occupied therefore could not compare with 2 locus occupied in each country.
(DOCX)

S5 Fig Histological activities according to the major genotype *babA/babB/-* (Ma) and other genotypes with *babA*-positive status (Mi). (DOCX)

S1 File Table A. Primer sequences for *bab* genotyping. Table B. *babA*-positive status and clinical diseases. Table C. Clinical diseases and *babA* at locus A versus *babA* at other locus (locus B and/or locus C). Table D. Clinical diseases according to one *babA* versus multiple (two or three) *babA*. Table E. Distribution of *bab* genotypes in all strains from different clinical outcomes. Table F. Comparision of histological scores between *babA/babB/-* and other genotypes. Table G. *cagA* and *vacA* positive status in major (*babA/babB/-)* and minor (other genotypes with *babA*-positive status) genotypes.
(XLSX)
