# Supplementary material for: Helicobacter pylori bab characterization in clinical isolates from Bhutan, Myanmar, Nepal and Bangladesh
Source: PLoS One. 2017 Nov 6;12(11):e0187225. doi: 10.1371/journal.pone.0187225 (PMC5673166; doi:10.1371/journal.pone.0187225)
Supplement: S1 Fig — (DOCX) [file pone.0187225.s001.docx]

**S1 Fig.** ***H. pylori babA*-positive status and histological activities.**
