# Supplementary material for: Helicobacter pylori bab characterization in clinical isolates from Bhutan, Myanmar, Nepal and Bangladesh
Source: PLoS One. 2017 Nov 6;12(11):e0187225. doi: 10.1371/journal.pone.0187225 (PMC5673166; doi:10.1371/journal.pone.0187225)
Supplement: S2 Fig — A- babA at locus A and B- babA at other locus than A. In Bhutan all strains but one contained babA at locus A, thus we could not evaluate. (DOCX) [file pone.0187225.s002.docx]

**S2 Fig. Genomic location of *babA* and histological activities.** A- *babA* at locus A and B- *babA* at other locus than A. In Bhutan all strains but one contained *babA* at locus A, thus we could not evaluate.
