# Supplementary material for: Helicobacter pylori bab characterization in clinical isolates from Bhutan, Myanmar, Nepal and Bangladesh
Source: PLoS One. 2017 Nov 6;12(11):e0187225. doi: 10.1371/journal.pone.0187225 (PMC5673166; doi:10.1371/journal.pone.0187225)
Supplement: S4 Fig — 1-one locus occupied, 2- two loci occupied and 3- three loci occupied. In Bhutan only 5 strains were with three loci occupied, in Myanmar only 3 strains were with three loci occupied, in Nepal only 2 strains were with one locus occupied and in Bangladesh there were only 6 strains with one locus occupied therefore could not compare with 2 locus occupied in each country. (DOCX) [file pone.0187225.s004.docx]

**S4 Fig. Number of locus occupied and histological activities.** 1-one locus occupied, 2- two loci occupied and 3- three loci occupied. In Bhutan only 5 strains were with three loci occupied, in Myanmar only 3 strains were with three loci occupied, in Nepal only 2 strains were with one locus occupied and in Bangladesh there were only 6 strains with one locus occupied therefore could not compare with 2 locus occupied in each country.
